# 

**Published:** 1891-11-28

**Authors:** 


PRICE ONE PENNY.

No. 270, Vol. XI.—November 28th, 1891.

[Registered at the General Post Office as a Newspaper for transmission in the United Kingdom and Abroad.]

WITH EXTRA NURSING SUPPLEMENT.
Per Annum, 6s. 6d., post free.
Monthly Parts, 6d.; post free, 8d.
Ditto per Annum, 8s., post free.

### THE HOSPITAL

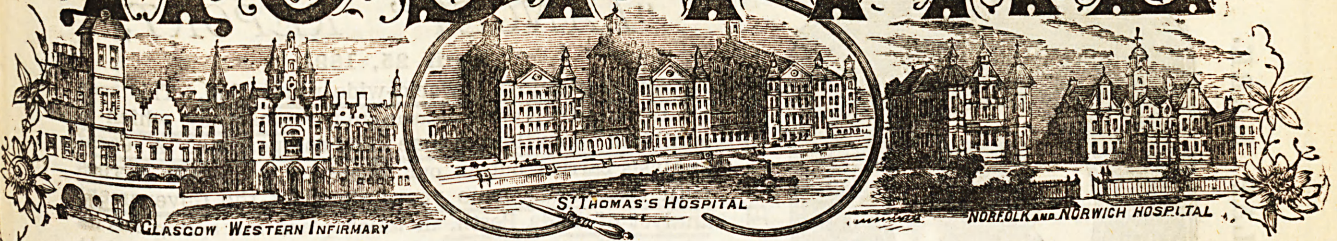

A WEEKLY JOURNAL OF

Science, Medicine, Nursing, and Philanthropy.

SATURDAY, NOVEMBER 28th, 1891.

A Weak Spot in the Popular Religion . . . . 97

Passing Topics.—"Save us from our Friends" . . . 98

The Doctor's Armchair.—The Woman or the Man? . . 99

The Most Interesting Hospital in the World . . 100

Some Scientific Aspects of Insanity. IV.—Consciousness and Mind . . . . 101

Notes and News . . . . 102

General Charities—Scraps and Gleanings . . . 103

Editor's Letter-Box.—Old Doctors or New? . . 103

Annotations.—More Light upon Inoculation—Where Should Hospitals be Placed?—The Scheduling of Poison . . . 104

The Practitioner's Mirror and Retrospect—Medicine.—Some Modern Theories of Gout . . . 105

A Model Hospital.—The New General Hospital at Eppendorf Hamburg . . . 106

The Student of Medicine.—Appointments—Vacancies—Notes from the Schools—Students' Medical Societies—Athletics

EXTRA SUPPLEMENT.—THE NURSING MIRROR.

En Passant.—Patients or Friends?—Kettering News—Cane Hill Asylum—Wolverhampton—British Nurses' Soiree—Doctors and Nurses—Short Items . . . xlix

Nursing Medals and Certificates . . . l

The Princess of Wales and the Nurses . . . l

Nursing in Denmark . . . l

Notes from Australia . . . li

For Reading to the Sick—The Blessing of Sickness . . li

A Nurse in Natal—Boots and Shoes . . . lii

A Bed for a Sick Nurse . . . liii

Notes and Queries . . . liii

Everybody's Opinion.—South Africa and English Nurses . liii

Appointments . . . liii

Wants and Workers . . . liii

Off Duty.—"Dr. Sutters" (continued) . . . liv

Amusements and Relaxation . . . liv

